# Effects of herbal ointment containing the leaf extracts of Madeira vine (*Anredera cordifolia* (Ten.) Steenis) for burn wound healing process on albino rats

**DOI:** 10.14202/vetworld.2017.808-813

**Published:** 2017-07-22

**Authors:** Wiwik Misaco Yuniarti, Bambang Sektiari Lukiswanto

**Affiliations:** Department of Clinical Science, Faculty of Veterinary Medicine, Universitas Airlangga, Mulyorejo, Kampus C Unair, Surabaya 60115, Indonesia

**Keywords:** burn wound, leaf extract of Madeira vine, wound healing

## Abstract

**Aim:**

Skin burn is a health problem that requires fast and accurate treatment. If not well-treated, the burn will cause various damaging conditions for the patient. The leaf extract of Madeira vine (*Anredera cordifolia* (Ten.) Steenis), or popularly known as Binahong in Indonesia, has been used to treat various diseases. The purpose of this research is to determine the effects of leaf extracts of Madeira vine (*A. cordifolia* (Ten.) Steenis) on skin burn healing process in rats as an animal model.

**Materials and Methods:**

In this research, there were four treatment groups: G0, G1, G2, and G3, each consisting of five rats. All these rats were given skin burns, using hot metal plates. Then, sulfadiazine was given to G0, 2.5% leaf extract of Madeira vine was given to G1, 5% extract was given to G2, and 10% extract was given to G3, for straight 14 days topically, 3 times a day. At the end of the treatment period, skin excisions were conducted, and histopathological examination was carried out.

**Result:**

Microscopic observation on the wound healing process on the collagen deposition, polymorphonuclear infiltration, angiogenesis, and fibrosis showed that G2 had a significant difference with G0, G1, and G3 (p<0.05), while group G0 was significantly different from G1 and G3 (p<0.05). The better burn healing process on G2 allegedly because of the activity of flavonoid, saponin, and tannin, contained in the Madeira vine, which have the antioxidant, anti-inflammatory, and antibacterial effects.

**Conclusion:**

The ointment from the 5% leaf extract of Madeira vine (*A. cordifolia* (Ten.) Steenis) has been proven to be effective to be used for topical burn therapy.

## Introduction

Burn is defined as destruction found in the epidermal tissue, dermal tissue, or deeper tissues, due to contact with thermal, chemical, or electrical agents [1]. Burn does not only cause skin damage, but this also affects the entire system of the patient’s body. In patients with extensive burns, their bodies might not be able to tolerate the condition any longer which causes various complications including death, and thus require special treatments [2].

World Health Organization estimates that every year there are approximately 265 thousand deaths caused by burns on humans. The incidence and burn-related death are 7 times higher in areas with low to middle per capita incomes, and almost half of these occurred in South East Asia [3].

The process of wound healing, including burn wound, can be divided into three phases; inflammation, proliferation, and remodeling [4]. The healing process of burn wound involves one of the key components in the wound healing phase, which is the formation of collagen. Collagen is the most found protein in human tissues, including in skin. Collagen also helps the hemostatic process, interacts with thrombocytes and fibronectin, and accelerates the cellular components and growth factor [5].

Burn wound treatment needs to be carried out as immediately as possible to prevent mild as well as severe complications, such as hypovolemic shock and sepsis. As from the cost factor, burn wound treatment is relatively expensive [6].

People commonly use herbal medication to heal various diseases because the cost is relatively low and it is easily accessible. Binahong or Madeira vine (*Anredera cordifolia* (Ten.) Steenis) is a herbal plant that is most frequently used to cure various kinds of diseases in a number of Asian countries, such as Vietnam, Taiwan, China, and Korea [7]. Several parts of this plant, particularly the leaves, are often used as herbal medicine [8]. Some people in Indonesia proved that the plant can treat diabetes mellitus, tuberculosis, rheumatic, uric acid, asthma, typhoid, hypertension, hemorrhoids, use as diuretic, postpartum recovery, wound healing and post-circumcision operating, gastritis, colitis, and cancer [7]. Another activity of this plant is as hepatoprotector, antiobesity, increase breast milk, and lowering blood pressure [9].

Madeira vine (*A. cordifolia* (Ten.) Steenis) leaves have such benefits as anti-inflammation, antioxidant, antibacterial, and analgesics [7,8]. The leaves contain bioactive compounds such as flavonoid, saponin, and tannin. The flavonoid in the leaves of Binahong has an anti-inflammation effect, while saponin works as an antiseptic that can terminate or prevent the growth of microorganism in the wound to avoid an infection, increase the number of fibroblast cells, and stimulate the formation of collagen [10].

The aim of this research is to determine the effects of the leaf extracts of Madeira vine (*A. cordifolia* (Ten.) Steenis) toward the wound healing process in rats (*Rattus norvegicus*) that suffered from burns. The result will then be compared to that of treatment with sulfadiazine in a standard burn wound therapy.

## Materials and Methods

### Ethical approval

The entire research was conducted appropriately following the ethics in using experimental animals and has been approved by the Ethics Commission of the Faculty of Veterinary Medicine, Universitas Airlangga.

### The leaf extract preparation of madeira vine

The leaves of Madeira vine (*A. cordifolia* (Ten.) Steenis) that had been picked, washed, drained, and chopped, were then dried by exposing them to direct sunlight. The drying process was then continued using an oven, heated at 40°C to reach complete dried leaves.

The dried leaves were then mashed by soaking them in a 96% ethanol solution with a ratio of 1:10, in accordance with the Indonesian Pharmacopoeia for 5 days in a measuring cup, and the soak was then stirred occasionally. After 5 days, the first debris and filtrate were separated with a filter paper. The first debris was then soaked again with a 96% ethanol solution for another 2 days and stirred occasionally. The second debris and filtrate were then separated with filter paper.

The first and second filtrates were *R. norvegicus* and filtered again to make sure that there was no debris to acquire totally macerated leaves. The filtrates were then evaporated using a vacuum evaporator at a temperature of 60°C to acquire almost thick extracts and then continued with a water bath at 60°C to get thick extracts.

### The leaf extract ointment preparation of madeira vine

After a thick extract had been acquired, the step was continued with the making of an ointment with the ointment bases, adeps lanae, and vaselin album. The ointment was made in a mortar and stamper that had been sterilized before. Adeps lanae (15 g) was put first in the mortar, then stirred slowly with a stamper until miscible. Vaselin album (85 g) was put in the mortar and stirred slowly at a constant speed until adeps lanae and vaselin albums were evenly mixed. Leaf extracts of the Madeira vine were added based on the following respective concentrations 2.5%, 5%, and 10% to each group, and then stirred to get a homogenous ointment [11].

### Burn wound making on rats

The rats were anesthetized with the combination of ketamine and diazepam (100 mg/kg body weight [BW]: 5 mg/kg BW). The rats’ backs were shaved in the size of 3 cm × 3 cm and smeared with betadine. After the anesthesia, the next process was the burn wound making. An iron plate with the size of 2.5 cm × 2.5 cm was soaked in 100°C boiling water for 5 min. The burns were made on the back of the rats near the vertebrae thoracalis by patching the plate on the rats back for 30 s [12].

### Burn treatment on the rats

A total of 20 male, white rats were put randomly in five groups, each receiving five repeated treatments. The types of treatment are:

- G0: Rats with skin burns, treated with silver sulfadiazine
- G1: Rats with skin burns, treated with extract ointment 2.5%
- G2: Rats with skin burns, treated with extract ointment 5%
- G3: Rats with skin burns, treated with extract ointment 10%.

The treatment was carried out topically by smearing the leaf extract ointment of Madeira vine and silver sulfadiazine using clean cotton buds. The treatment was conducted 3 times daily with an interval of 8 h between treatments for 14 days [12].

### The histopathological slides preparation

The 3 cm × 3 cm excision on the white rats was conducted at the end of the experiment and was continued with the making of the histopathological slides. The histological parameters (polymorphonuclear [PMN] infiltration, collagen deposition, fibrosis, and angiogenesis) on the wound biopsy specimens were determined. The specimens were colored with Masson’s trichrome. The histological criteria are as follows: Collagen deposition score – Normal bundle: Score=2, unorganized/edema: Score=1, amorphous: Score=0; number of PMN: 0-10: Score=2, 11-40: Score=1, >40: Score=0; degree of angiogenesis, namely, mild, medium, and severe, and degree of fibrosis, by measuring the collagen bundles thickness in three degrees, namely, mild, medium, and severe [13].

## Results

**Figure-1:**
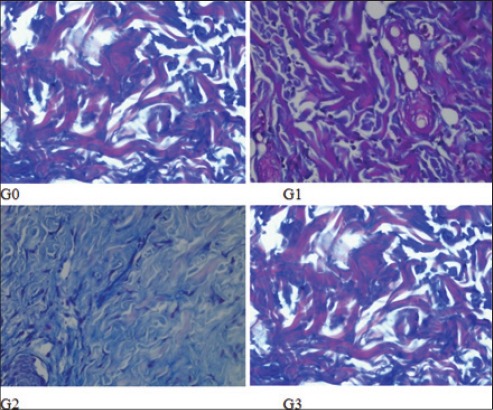
Collagen deposition on each group. Collagen deposition on G2 has the highest score compared to other groups.

**Figure-2:**
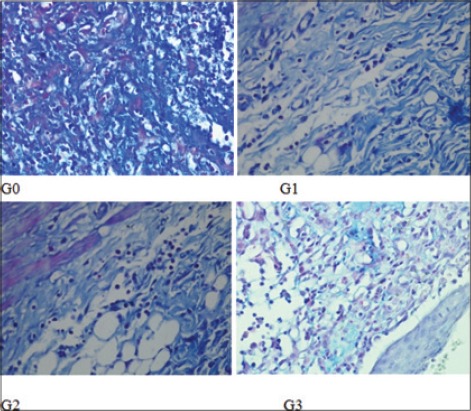
Polymorphonuclear (PMN) cell infiltration on each group. PMN infiltration on G2 has the highest score compared to other groups.

**Figure-3:**
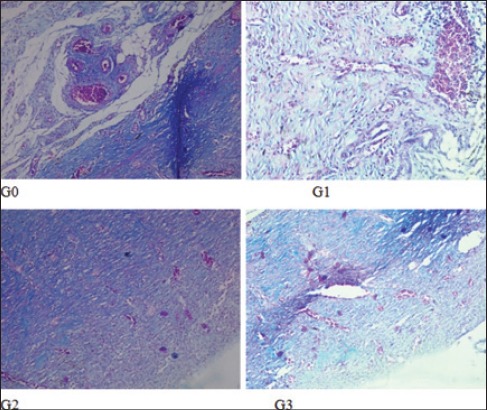
Degree of angiogenesis on each group. Degree of angiogenesis on G2 lighter (mild) than other groups (medium).

**Figure-4:**
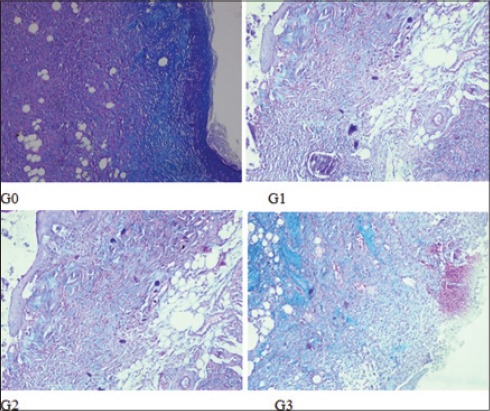
Degree of fibrosis on each group. Degree of fibrosis on G2 was at the medium level; while on the other groups were mild.

The result variable of this research is the collagen deposition and PMN score, degree of angiogenesis and fibrosis (Table-1). The collagen deposition score and PMN score (2.0±0.00 and 2.0±0.00) on G2 are highest and accompanied with mild angiogenesis and medium fibrosis. On the G1 and G3, the collagen deposition and PMN score (1.4±0.55 and 1.6±0.55; 1.6±0.55 and 1.6±0.55) not as good as G2 with moderate angiogenesis and mild fibrosis. The same conditions are seen in G0, but with lowest collagen deposition and PMN score (1.0±0.00 and 1.4±0.55) (Figures-1-4).

**Table-1:** Result of collagen deposition, PMN score, angiogenesis, and fibrosis.

Group	Cycles	Collagen deposition score	PMN score	Degree of angiogenesis	Degree of fibrosis
G0	1	1	1	Medium	Mild
	2	1	1	Medium	Mild
	3	1	1	Medium	Mild
	4	1	2	Medium	Mild
	5	1	2	Medium	Mild
G1	1	1	2	Medium	Mild
	2	2	1	Medium	Mild
	3	2	2	Medium	Mild
	4	1	1	Medium	Mild
	5	1	2	Medium	Mild
G2	1	2	2	Mild	Medium
	2	2	2	Mild	Medium
	3	2	2	Mild	Medium
	4	2	2	Mild	Medium
	5	2	2	Mild	Medium
G3	1	2	1	Medium	Mild
	2	2	1	Medium	Mild
	3	2	2	Medium	Mild
	4	1	2	Medium	Mild
	5	1	2	Medium	Mild

PMN=Polymorphonuclear

The result of this research showed that through observation and analysis of the wound healing indicators, comprise collagen deposition, PMN infiltration, angiogenesis, and fibrosis, G2 showed a significant difference from G0, G1, and G3 (p<0.05), and G0 was significantly different from G1 and G3 (p<0.05) (Table-2). The use leaf extract ointment of Madeira vine showed a better result in the burn healing process than the use of silver sulfadiazine.

**Table-2:** Comparison of healing processes in each treatment.

Treatment	Collagen deposition score	PMN score	Degree of angiogenesis	Degree fibrosis
G0	1.0^c^±0.00	1.4^c^±0.55	Medium	Mild
G1	1.4^b^±0.55	1.6^b^±0.55	Medium	Mild
G2	2.0^a^±0.00	2.0^a^±0.00	Mild	Medium
G3	1.6^b^±0.55	1.6^b^±0.55	Medium	Mild

Values are expressed as the mean of five individuals in each group±SD. Means with different superscripts letters are significant at p<0.05. SD=Standard deviation, PMN=Polymorphonuclear

The burn healing process is similar with the wound healing process in general, which is through the phases of inflammation, proliferation, and remodeling. This normal healing process can be obstructed in each of those phases, depending on the factors that influence the process. Various studies have proven that infection is the main cause for healing failure and can even cause mortality in burn patients [14]. Therefore, a lot of research has been conducted to prevent infection and to accelerate the healing process, one of which is through the use of topical antibiotics which has been proven effective in preventing death [15].

Silver Sulfadiazine ointment is the golden standard in the treatment of burn patients. Some of the reasons are because this ointment is easily applied, easy to find, does not hurt when being applied, has low toxicity, and has antibacterial effects [12]. Silver sulfadiazine has a positive effect in the proliferation of fibroblasts which produce collagen and fibronectin. This cream is able to stimulate cells such as macrophage to produce growth factor and cytokines during the wound healing process [16]. Nonetheless, silver sulfadiazine also has side effects, such as neutropenia, erythema multiforme, crystalluria, methemoglobinemia, and wound healing process delay [17]. These are the reasons why the wound healing process in G0 was not as good as those in other treatment groups.

In G1 (2.5% extract) and G3 (10% extract), the results showed that the healing profiles were not as good as those in G2. Humidity was one of the conditions affected the wound healing process. Humidity has a permeability character with oxygen and water vapor, but it is occlusive against bacteria and water, so the healing process is not affected [18]. Oxygen is a vital nutrition for cell metabolism and is extremely required in wound healing process [19].

The giving of the 2.5% leaf extract in G1 caused higher humidity level on the wound surface compared to the humidity levels on G2 (5% extract) and G3 (10% extract). This relatively high humidity level caused a high level of oxygen in the wound tissue. This condition can stimulated the deposition of collagen since the proliferation process of fibroblasts was increase [20].

In G3 (10% extract), the humidity level was lower than those in G1 (2.5% extract) and G2 (5% extract). The low humidity would cause decrease of oxygen pressure on the wound tissue, affecting the functions of neutrophils, macrophages, and fibroblasts, and obstruct the collagen synthesis process [19,21]. This is why at a 10% concentration, the wound healing process did not happen quite well [22].

It was found that the process of wound healing in G2 (5% extract) is better than the other groups. This result showed that the leaf extract of Madeira vine with a 5% concentration is an optimum dose that can fasten the wound healing process. This is connected to the secondary metabolites of Madeira vine that function as medicine. These metabolites are flavonoid, saponin, tannin, and ascorbic acid [23].

Flavonoid has the ability to be an antioxidant that can reduce free radicals. The antioxidant will bond with free radicals that damage cell membranes, making the cells unable to function perfectly. With this bond, damages in the cell membranes can be reduced and the proliferation phase in wound healing can occur expectedly well [24].

Flavonoid can also function as a destroyer of microbes, especially the Gram-negative bacteria. Flavonoid has a mechanism as an anti-bacterium by forming complex compounds with dissolved and extracellular proteins that can damage bacteria’s cell wall, which is followed by the release of intracellular compounds [24].

Saponin can increase the proliferation of monocytes which eventually will increase the number of macrophages that secrete the growth factor which is crucial for the wound healing process. Saponin can also fasten the migration process of keratinocytes which have an important role in the process of reepithelialization [25].

Tannin in the Madeira vine leaves functions as an astringent that can cause skin pores to shrink and stop exudates and mild bleeding, causing wound to close and preventing bleeding on the wound [26]. Tannin and saponin have a role in the migration and proliferation of fibroblasts in wound, making wound contract faster [25,26]. Ascorbic acid is important in activating prolyl hydroxylase that supports the hydroxylation stage in collagen deposition [27].

Improvement in the wound healing process was suspected to be because 5% Madeira vine or Binahong, has an antioxidant, anti-inflammation, and antibacterial activities. This is shown by the acceleration in the wound healing process, marked with the low number of PMN, high quality of collagen, and mild angiogenesis, and medium fibrosis. This condition is even better compared to the treatment group which received silver sulfadiazine as the standard therapy for burn patients.

## Conclusion

The burn treatment using 5% leaf extracts ointment of Madeira vine (*A. cordifolia* (Ten.) Steenis) was proven to accelerate the healing process of burn wound in the skins of white rats (*R. norvegicus*).

## Authors’ Contributions

WMY and BSL carried out the main research works, performed the statistical analysis and analyzed the main data in the experiments and approved the final manuscript.
